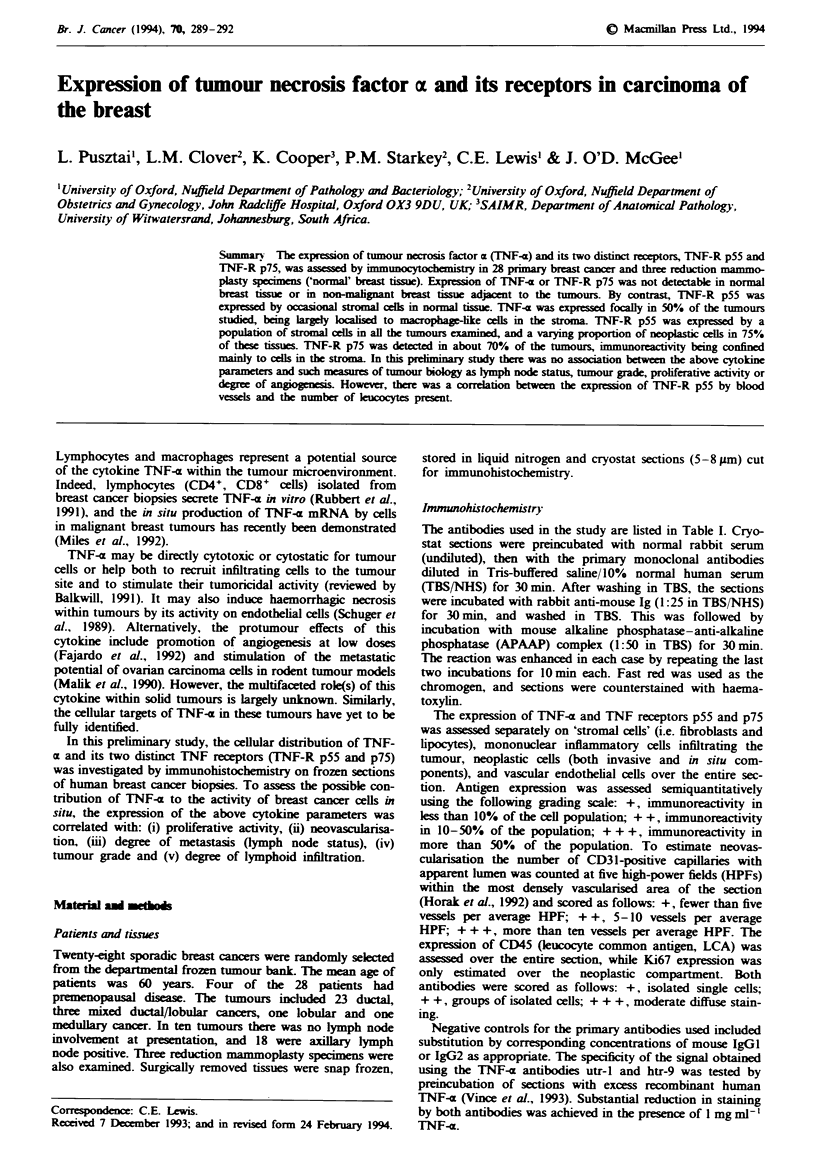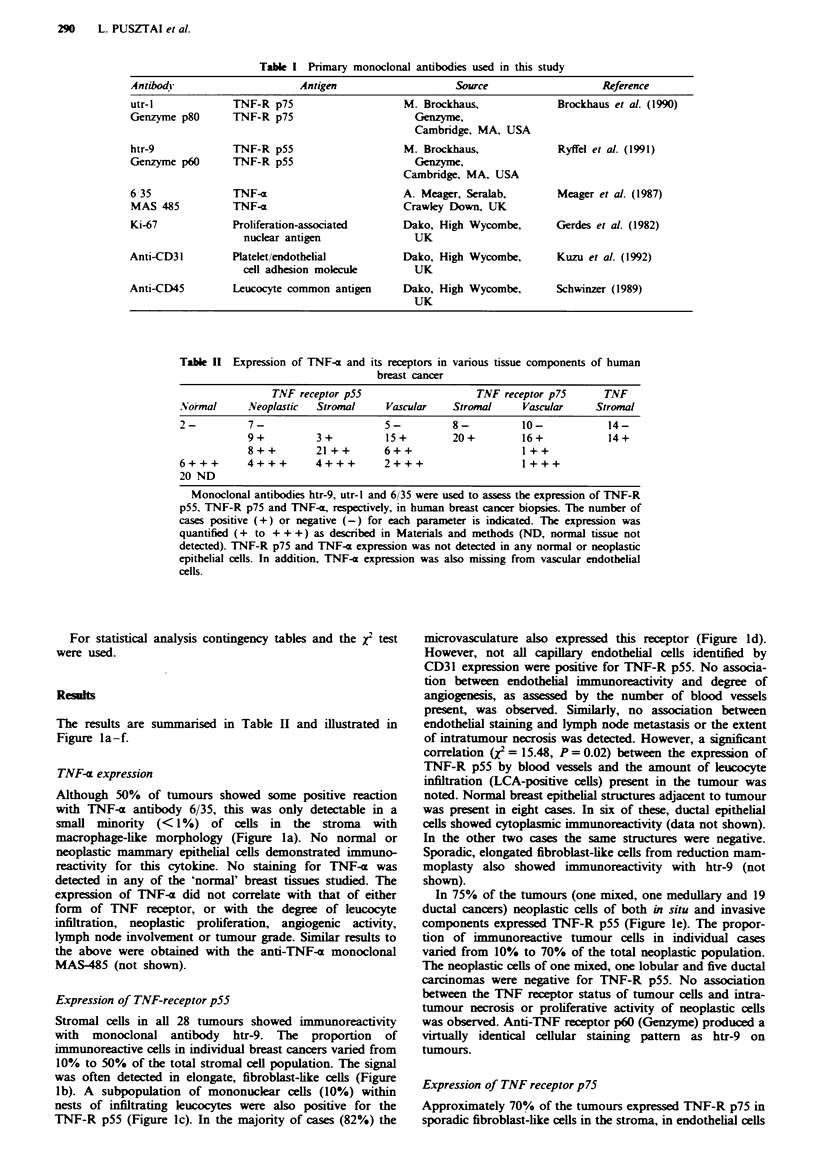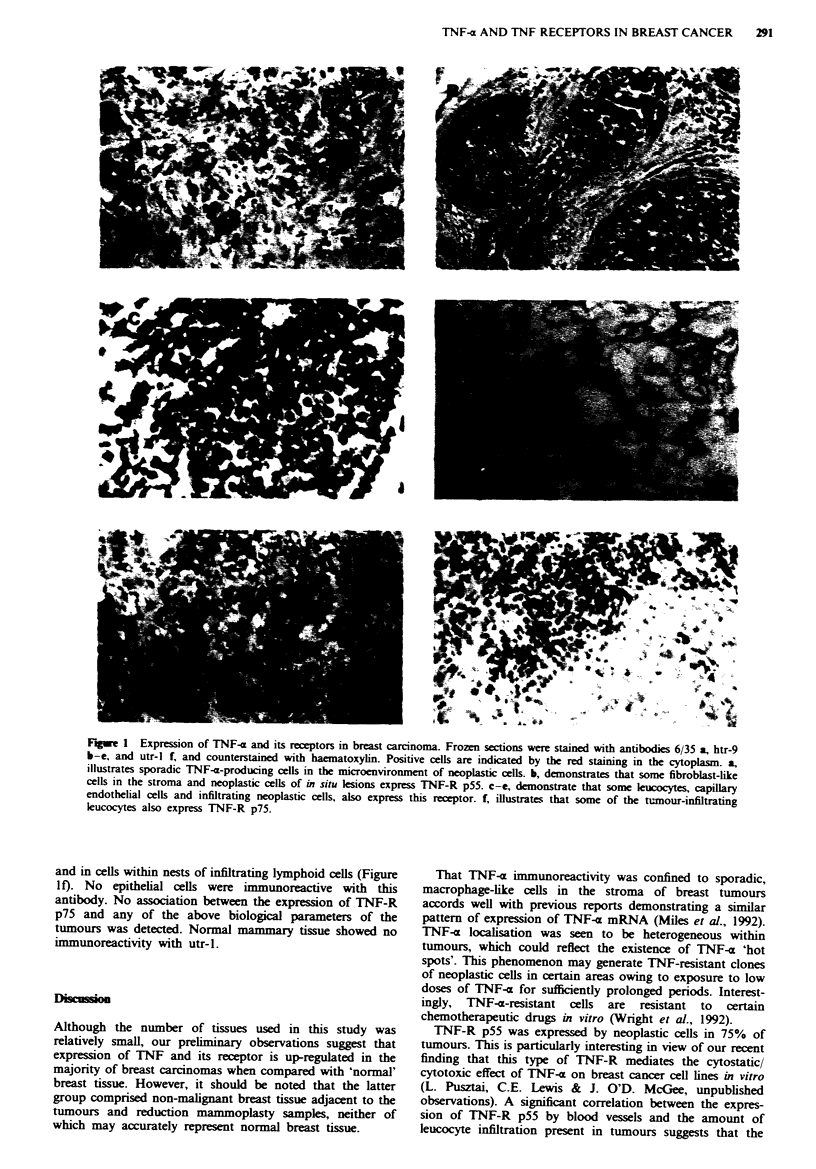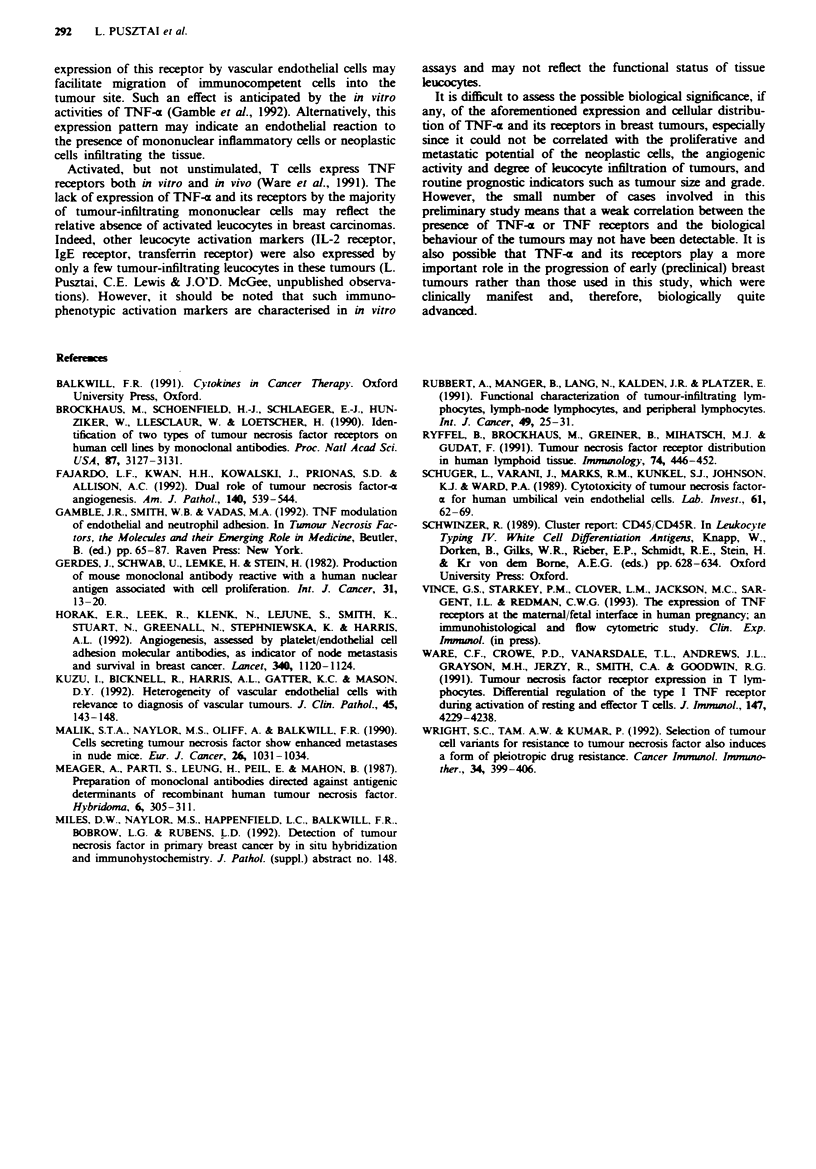# Expression of tumour necrosis factor alpha and its receptors in carcinoma of the breast.

**DOI:** 10.1038/bjc.1994.294

**Published:** 1994-08

**Authors:** L. Pusztai, L. M. Clover, K. Cooper, P. M. Starkey, C. E. Lewis, J. O. McGee

**Affiliations:** University of Oxford, Nuffield Department of Pathology and Bacteriology, UK.

## Abstract

**Images:**


Br. J. Cancer (1994), 70, 289-292 © Macmillan Press Ltd., 1994

### Expression of tumour necrosis factor α and its receptors in carcinoma of the breast

L. Pusztai[1], L.M. Clover[2], K. Cooper[3], P.M. Starkey[2], C.E. Lewis[1] & J. O'D. McGee[1]

[1]*University of Oxford, Nuffield Department of Pathology and Bacteriology;* [2]*University of Oxford, Nuffield Department of Obstetrics and Gynecology, John Radcliffe Hospital, Oxford OX3 9DU, UK;* [3]*SAIMR, Department of Anatomical Pathology, University of Witwatersrand, Johannesburg, South Africa.*

Summary The expression of tumour necrosis factor α (TNF-α) and its two distinct receptors, TNF-R p55 and TNF-R p75, was assessed by immunocytochemistry in 28 primary breast cancer and three reduction mammoplasty specimens ('normal' breast tissue). Expression of TNF-α or TNF-R p75 was not detectable in normal breast tissue or in non-malignant breast tissue adjacent to the tumours. By contrast, TNF-R p55 was expressed by occasional stromal cells in normal tissue. TNF-α was expressed focally in 50% of the tumours studied, being largely localised to macrophage-like cells in the stroma. TNF-R p55 was expressed by a population of stromal cells in all the tumours examined, and a varying proportion of neoplastic cells in 75% of these tissues. TNF-R p75 was detected in about 70% of the tumours, immunoreactivity being confined mainly to cells in the stroma. In this preliminary study there was no association between the above cytokine parameters and such measures of tumour biology as lymph node status, tumour grade, proliferative activity or degree of angiogenesis. However, there was a correlation between the expression of TNF-R p55 by blood vessels and the number of leucocytes present.

Lymphocytes and macrophages represent a potential source of the cytokine TNF-α within the tumour microenvironment. Indeed, lymphocytes ($CD4^+$, $CD8^+$ cells) isolated from breast cancer biopsies secrete TNF-α *in vitro* (Rubbert *et al.*, 1991), and the *in situ* production of TNF-α mRNA by cells in malignant breast tumours has recently been demonstrated (Miles *et al.*, 1992).

TNF-α may be directly cytotoxic or cytostatic for tumour cells or help both to recruit infiltrating cells to the tumour site and to stimulate their tumoricidal activity (reviewed by Balkwill, 1991). It may also induce haemorrhagic necrosis within tumours by its activity on endothelial cells (Schuger *et al.*, 1989). Alternatively, the protumour effects of this cytokine include promotion of angiogenesis at low doses (Fajardo *et al.*, 1992) and stimulation of the metastatic potential of ovarian carcinoma cells in rodent tumour models (Malik *et al.*, 1990). However, the multifaceted role(s) of this cytokine within solid tumours is largely unknown. Similarly, the cellular targets of TNF-α in these tumours have yet to be fully identified.

In this preliminary study, the cellular distribution of TNF-α and its two distinct TNF receptors (TNF-R p55 and p75) was investigated by immunohistochemistry on frozen sections of human breast cancer biopsies. To assess the possible contribution of TNF-α to the activity of breast cancer cells *in situ*, the expression of the above cytokine parameters was correlated with: (i) proliferative activity, (ii) neovascularisation, (iii) degree of metastasis (lymph node status), (iv) tumour grade and (v) degree of lymphoid infiltration.

### Material and methods

### *Patients and tissues*

Twenty-eight sporadic breast cancers were randomly selected from the departmental frozen tumour bank. The mean age of patients was 60 years. Four of the 28 patients had premenopausal disease. The tumours included 23 ductal, three mixed ductal/lobular cancers, one lobular and one medullary cancer. In ten tumours there was no lymph node involvement at presentation, and 18 were axillary lymph node positive. Three reduction mammoplasty specimens were also examined. Surgically removed tissues were snap frozen, stored in liquid nitrogen and cryostat sections (5-8 μm) cut for immunohistochemistry.

Correspondence: C.E. Lewis.

Received 7 December 1993; and in revised form 24 February 1994.

### *Immunohistochemistry*

The antibodies used in the study are listed in Table I. Cryostat sections were preincubated with normal rabbit serum (undiluted), then with the primary monoclonal antibodies diluted in Tris-buffered saline/10% normal human serum (TBS/NHS) for 30 min. After washing in TBS, the sections were incubated with rabbit anti-mouse Ig (1:25 in TBS/NHS) for 30 min, and washed in TBS. This was followed by incubation with mouse alkaline phosphatase-anti-alkaline phosphatase (APAAP) complex (1:50 in TBS) for 30 min. The reaction was enhanced in each case by repeating the last two incubations for 10 min each. Fast red was used as the chromogen, and sections were counterstained with haematoxylin.

The expression of TNF-α and TNF receptors p55 and p75 was assessed separately on 'stromal cells' (i.e. fibroblasts and lipocytes), mononuclear inflammatory cells infiltrating the tumour, neoplastic cells (both invasive and *in situ* components), and vascular endothelial cells over the entire section. Antigen expression was assessed semiquantitatively using the following grading scale: +, immunoreactivity in less than 10% of the cell population; + +, immunoreactivity in 10-50% of the population; + + +, immunoreactivity in more than 50% of the population. To estimate neovascularisation the number of CD31-positive capillaries with apparent lumen was counted at five high-power fields (HPFs) within the most densely vascularised area of the section (Horak *et al.*, 1992) and scored as follows: +, fewer than five vessels per average HPF; + +, 5-10 vessels per average HPF; + + +, more than ten vessels per average HPF. The expression of CD45 (leucocyte common antigen, LCA) was assessed over the entire section, while Ki67 expression was only estimated over the neoplastic compartment. Both antibodies were scored as follows: +, isolated single cells; + +, groups of isolated cells; + + +, moderate diffuse staining.

Negative controls for the primary antibodies used included substitution by corresponding concentrations of mouse IgG1 or IgG2 as appropriate. The specificity of the signal obtained using the TNF-α antibodies utr-1 and htr-9 was tested by preincubation of sections with excess recombinant human TNF-α (Vince *et al.*, 1993). Substantial reduction in staining by both antibodies was achieved in the presence of 1 mg $ml^{-1}$ TNF-α.

**Table I** Primary monoclonal antibodies used in this study

| *Antibody* | *Antigen* | *Source* | *Reference* |
|---|---|---|---|
| utr-1 | TNF-R p75 | M. Brockhaus, | Brockhaus *et al.* (1990) |
| Genzyme p80 | TNF-R p75 | Genzyme, Cambridge, MA, USA | |
| htr-9 | TNF-R p55 | M. Brockhaus, | Ryffel *et al.* (1991) |
| Genzyme p60 | TNF-R p55 | Genzyme, Cambridge, MA, USA | |
| 6/35 | TNF-α | A. Meager, Seralab, | Meager *et al.* (1987) |
| MAS 485 | TNF-α | Crawley Down, UK | |
| Ki-67 | Proliferation-associated nuclear antigen | Dako, High Wycombe, UK | Gerdes *et al.* (1982) |
| Anti-CD31 | Platelet/endothelial cell adhesion molecule | Dako, High Wycombe, UK | Kuzu *et al.* (1992) |
| Anti-CD45 | Leucocyte common antigen | Dako, High Wycombe, UK | Schwinzer (1989) |

**Table II** Expression of TNF-α and its receptors in various tissue components of human breast cancer

| | *TNF receptor p55* | | | | *TNF receptor p75* | | *TNF* |
|---|---|---|---|---|---|---|---|
| *Normal* | | *Neoplastic* | *Stromal* | *Vascular* | *Stromal* | *Vascular* | *Stromal* |
| 2 − | | 7 − | | 5 − | 8 − | 10 − | 14 − |
| | | 9 + | 3 + | 15 + | 20 + | 16 + | 14 + |
| | | 8 + + | 21 + + | 6 + + | | 1 + + | |
| 6 + + + | | 4 + + + | 4 + + + | 2 + + + | | 1 + + + | |
| 20 ND | | | | | | | |

Monoclonal antibodies htr-9, utr-1 and 6/35 were used to assess the expression of TNF-R p55, TNF-R p75 and TNF-α, respectively, in human breast cancer biopsies. The number of cases positive (+) or negative (−) for each parameter is indicated. The expression was quantified (+ to + + +) as described in Materials and methods (ND, normal tissue not detected). TNF-R p75 and TNF-α expression was not detected in any normal or neoplastic epithelial cells. In addition, TNF-α expression was also missing from vascular endothelial cells.

For statistical analysis contingency tables and the $\chi^2$ test were used.

### Results

The results are summarised in Table II and illustrated in Figure 1a–f.

### *TNF-α expression*

Although 50% of tumours showed some positive reaction with TNF-α antibody 6/35, this was only detectable in a small minority (<1%) of cells in the stroma with macrophage-like morphology (Figure 1a). No normal or neoplastic mammary epithelial cells demonstrated immunoreactivity for this cytokine. No staining for TNF-α was detected in any of the 'normal' breast tissues studied. The expression of TNF-α did not correlate with that of either form of TNF receptor, or with the degree of leucocyte infiltration, neoplastic proliferation, angiogenic activity, lymph node involvement or tumour grade. Similar results to the above were obtained with the anti-TNF-α monoclonal MAS-485 (not shown).

### *Expression of TNF-receptor p55*

Stromal cells in all 28 tumours showed immunoreactivity with monoclonal antibody htr-9. The proportion of immunoreactive cells in individual breast cancers varied from 10% to 50% of the total stromal cell population. The signal was often detected in elongate, fibroblast-like cells (Figure 1b). A subpopulation of mononuclear cells (10%) within nests of infiltrating leucocytes were also positive for the TNF-R p55 (Figure 1c). In the majority of cases (82%) the microvasculature also expressed this receptor (Figure 1d). However, not all capillary endothelial cells identified by CD31 expression were positive for TNF-R p55. No association between endothelial immunoreactivity and degree of angiogenesis, as assessed by the number of blood vessels present, was observed. Similarly, no association between endothelial staining and lymph node metastasis or the extent of intratumour necrosis was detected. However, a significant correlation ($\chi^2 = 15.48$, $P = 0.02$) between the expression of TNF-R p55 by blood vessels and the amount of leucocyte infiltration (LCA-positive cells) present in the tumour was noted. Normal breast epithelial structures adjacent to tumour was present in eight cases. In six of these, ductal epithelial cells showed cytoplasmic immunoreactivity (data not shown). In the other two cases the same structures were negative. Sporadic, elongated fibroblast-like cells from reduction mammoplasty also showed immunoreactivity with htr-9 (not shown).

In 75% of the tumours (one mixed, one medullary and 19 ductal cancers) neoplastic cells of both *in situ* and invasive components expressed TNF-R p55 (Figure 1e). The proportion of immunoreactive tumour cells in individual cases varied from 10% to 70% of the total neoplastic population. The neoplastic cells of one mixed, one lobular and five ductal carcinomas were negative for TNF-R p55. No association between the TNF receptor status of tumour cells and intratumour necrosis or proliferative activity of neoplastic cells was observed. Anti-TNF receptor p60 (Genzyme) produced a virtually identical cellular staining pattern as htr-9 on tumours.

### *Expression of TNF receptor p75*

Approximately 70% of the tumours expressed TNF-R p75 in sporadic fibroblast-like cells in the stroma, in endothelial cells

**Figure 1** Expression of TNF-α and its receptors in breast carcinoma. Frozen sections were stained with antibodies 6/35 **a**, htr-9 **b**–**e**, and utr-1 **f**, and counterstained with haematoxylin. Positive cells are indicated by the red staining in the cytoplasm. **a**, illustrates sporadic TNF-α-producing cells in the microenvironment of neoplastic cells. **b**, demonstrates that some fibroblast-like cells in the stroma and neoplastic cells of *in situ* lesions express TNF-R p55. **c**–**e**, demonstrate that some leucocytes, capillary endothelial cells and infiltrating neoplastic cells, also express this receptor. **f**, illustrates that some of the tumour-infiltrating leucocytes also express TNF-R p75.

and in cells within nests of infiltrating lymphoid cells (Figure 1f). No epithelial cells were immunoreactive with this antibody. No association between the expression of TNF-R p75 and any of the above biological parameters of the tumours was detected. Normal mammary tissue showed no immunoreactivity with utr-1.

### Discussion

Although the number of tissues used in this study was relatively small, our preliminary observations suggest that expression of TNF and its receptor is up-regulated in the majority of breast carcinomas when compared with 'normal' breast tissue. However, it should be noted that the latter group comprised non-malignant breast tissue adjacent to the tumours and reduction mammoplasty samples, neither of which may accurately represent normal breast tissue.

That TNF-α immunoreactivity was confined to sporadic, macrophage-like cells in the stroma of breast tumours accords well with previous reports demonstrating a similar pattern of expression of TNF-α mRNA (Miles *et al*., 1992). TNF-α localisation was seen to be heterogeneous within tumours, which could reflect the existence of TNF-α 'hot spots'. This phenomenon may generate TNF-resistant clones of neoplastic cells in certain areas owing to exposure to low doses of TNF-α for sufficiently prolonged periods. Interestingly, TNF-α-resistant cells are resistant to certain chemotherapeutic drugs *in vitro* (Wright *et al*., 1992).

TNF-R p55 was expressed by neoplastic cells in 75% of tumours. This is particularly interesting in view of our recent finding that this type of TNF-R mediates the cytostatic/cytotoxic effect of TNF-α on breast cancer cell lines *in vitro* (L. Pusztai, C.E. Lewis & J. O'D. McGee, unpublished observations). A significant correlation between the expression of TNF-R p55 by blood vessels and the amount of leucocyte infiltration present in tumours suggests that the expression of this receptor by vascular endothelial cells may facilitate migration of immunocompetent cells into the tumour site. Such an effect is anticipated by the *in vitro* activities of TNF-α (Gamble *et al.*, 1992). Alternatively, this expression pattern may indicate an endothelial reaction to the presence of mononuclear inflammatory cells or neoplastic cells infiltrating the tissue.

Activated, but not unstimulated, T cells express TNF receptors both *in vitro* and *in vivo* (Ware *et al.*, 1991). The lack of expression of TNF-α and its receptors by the majority of tumour-infiltrating mononuclear cells may reflect the relative absence of activated leucocytes in breast carcinomas. Indeed, other leucocyte activation markers (IL-2 receptor, IgE receptor, transferrin receptor) were also expressed by only a few tumour-infiltrating leucocytes in these tumours (L. Pusztai, C.E. Lewis & J.O'D. McGee, unpublished observations). However, it should be noted that such immunophenotypic activation markers are characterised in *in vitro* assays and may not reflect the functional status of tissue leucocytes.

It is difficult to assess the possible biological significance, if any, of the aforementioned expression and cellular distribution of TNF-α and its receptors in breast tumours, especially since it could not be correlated with the proliferative and metastatic potential of the neoplastic cells, the angiogenic activity and degree of leucocyte infiltration of tumours, and routine prognostic indicators such as tumour size and grade. However, the small number of cases involved in this preliminary study means that a weak correlation between the presence of TNF-α or TNF receptors and the biological behaviour of the tumours may not have been detectable. It is also possible that TNF-α and its receptors play a more important role in the progression of early (preclinical) breast tumours rather than those used in this study, which were clinically manifest and, therefore, biologically quite advanced.

### References

BALKWILL, F.R. (1991). *Cytokines in Cancer Therapy*. Oxford University Press, Oxford.

BROCKHAUS, M., SCHOENFIELD, H.-J., SCHLAEGER, E.-J., HUNZIKER, W., LLESCLAUR, W. & LOETSCHER, H. (1990). Identification of two types of tumour necrosis factor receptors on human cell lines by monoclonal antibodies. *Proc. Natl Acad Sci. USA*, **87**, 3127–3131.

FAJARDO, L.F., KWAN, H.H., KOWALSKI, J., PRIONAS, S.D. & ALLISON, A.C. (1992). Dual role of tumour necrosis factor-α angiogenesis. *Am. J. Pathol.*, **140**, 539–544.

GAMBLE, J.R., SMITH, W.B. & VADAS, M.A. (1992). TNF modulation of endothelial and neutrophil adhesion. In *Tumour Necrosis Factors, the Molecules and their Emerging Role in Medicine*, Beutler, B. (ed.) pp. 65–87. Raven Press: New York.

GERDES, J., SCHWAB, U., LEMKE, H. & STEIN, H. (1982). Production of mouse monoclonal antibody reactive with a human nuclear antigen associated with cell proliferation. *Int. J. Cancer*, **31**, 13–20.

HORAK, E.R., LEEK, R., KLENK, N., LEJUNE, S., SMITH, K., STUART, N., GREENALL, N., STEPHNIEWSKA, K. & HARRIS, A.L. (1992). Angiogenesis, assessed by platelet/endothelial cell adhesion molecular antibodies, as indicator of node metastasis and survival in breast cancer. *Lancet*, **340**, 1120–1124.

KUZU, I., BICKNELL, R., HARRIS, A.L., GATTER, K.C. & MASON, D.Y. (1992). Heterogeneity of vascular endothelial cells with relevance to diagnosis of vascular tumours. *J. Clin. Pathol.*, **45**, 143–148.

MALIK, S.T.A., NAYLOR, M.S., OLIFF, A. & BALKWILL, F.R. (1990). Cells secreting tumour necrosis factor show enhanced metastases in nude mice. *Eur. J. Cancer*, **26**, 1031–1034.

MEAGER, A., PARTI, S., LEUNG, H., PEIL, E. & MAHON, B. (1987). Preparation of monoclonal antibodies directed against antigenic determinants of recombinant human tumour necrosis factor. *Hybridoma*, **6**, 305–311.

MILES, D.W., NAYLOR, M.S., HAPPENFIELD, L.C., BALKWILL, F.R., BOBROW, L.G. & RUBENS, L.D. (1992). Detection of tumour necrosis factor in primary breast cancer by in situ hybridization and immunohystochemistry. *J. Pathol.* (suppl.) abstract no. 148.

RUBBERT, A., MANGER, B., LANG, N., KALDEN, J.R. & PLATZER, E. (1991). Functional characterization of tumour-infiltrating lymphocytes, lymph-node lymphocytes, and peripheral lymphocytes. *Int. J. Cancer*, **49**, 25–31.

RYFFEL, B., BROCKHAUS, M., GREINER, B., MIHATSCH, M.J. & GUDAT, F. (1991). Tumour necrosis factor receptor distribution in human lymphoid tissue. *Immunology*, **74**, 446–452.

SCHUGER, L., VARANI, J., MARKS, R.M., KUNKEL, S.J., JOHNSON, K.J. & WARD, P.A. (1989). Cytotoxicity of tumour necrosis factor-α for human umbilical vein endothelial cells. *Lab. Invest.*, **61**, 62–69.

SCHWINZER, R. (1989). Cluster report: CD45/CD45R. In *Leukocyte Typing IV. White Cell Differentiation Antigens*, Knapp, W., Dorken, B., Gilks, W.R., Rieber, E.P., Schmidt, R.E., Stein, H. & Kr von dem Borne, A.E.G. (eds.) pp. 628–634. Oxford University Press: Oxford.

VINCE, G.S., STARKEY, P.M., CLOVER, L.M., JACKSON, M.C., SARGENT, I.L. & REDMAN, C.W.G. (1993). The expression of TNF receptors at the maternal/fetal interface in human pregnancy; an immunohistological and flow cytometric study. *Clin. Exp. Immunol.* (in press).

WARE, C.F., CROWE, P.D., VANARSDALE, T.L., ANDREWS, J.L., GRAYSON, M.H., JERZY, R., SMITH, C.A. & GOODWIN, R.G. (1991). Tumour necrosis factor receptor expression in T lymphocytes. Differential regulation of the type I TNF receptor during activation of resting and effector T cells. *J. Immunol.*, **147**, 4229–4238.

WRIGHT, S.C., TAM, A.W. & KUMAR, P. (1992). Selection of tumour cell variants for resistance to tumour necrosis factor also induces a form of pleiotropic drug resistance. *Cancer Immunol. Immunother.*, **34**, 399–406.